# A numerical study of different projection-based model reduction techniques applied to computational homogenisation

**DOI:** 10.1007/s00466-017-1428-x

**Published:** 2017-06-08

**Authors:** Dominic Soldner, Benjamin Brands, Reza Zabihyan, Paul Steinmann, Julia Mergheim

**Affiliations:** Chair of Applied Mechanics, Friedrich-Alexander-Universität Erlangen-Nürnberg, Egerlandstrasse 5, 91058 Erlangen, Germany

**Keywords:** Computational homogenisation, Hyper-reduction, Reduced-order modelling, Hyperelasticity

## Abstract

Computing the macroscopic material response of a continuum body commonly involves the formulation of a phenomenological constitutive model. However, the response is mainly influenced by the heterogeneous microstructure. Computational homogenisation can be used to determine the constitutive behaviour on the macro-scale by solving a boundary value problem at the micro-scale for every so-called macroscopic material point within a nested solution scheme. Hence, this procedure requires the repeated solution of similar microscopic boundary value problems. To reduce the computational cost, model order reduction techniques can be applied. An important aspect thereby is the robustness of the obtained reduced model. Within this study reduced-order modelling (ROM) for the geometrically nonlinear case using hyperelastic materials is applied for the boundary value problem on the micro-scale. This involves the Proper Orthogonal Decomposition (POD) for the primary unknown and hyper-reduction methods for the arising nonlinearity. Therein three methods for hyper-reduction, differing in how the nonlinearity is approximated and the subsequent projection, are compared in terms of accuracy and robustness. Introducing interpolation or Gappy-POD based approximations may not preserve the symmetry of the system tangent, rendering the widely used Galerkin projection sub-optimal. Hence, a different projection related to a Gauss-Newton scheme (Gauss-Newton with Approximated Tensors- GNAT) is favoured to obtain an optimal projection and a robust reduced model.

## Introduction

Phenomenological constitutive models are frequently used to compute the material response of a continuum body. However, the main influence of the macroscopic response is driven by the heterogeneous microstructure, whereas the direct modelling of the underlying microstructure is usually not feasible. A variety of analytical methods exists to account for the microstructure, e. g. Eshelby  [12] or Mori-Tanaka-Method  [27]. Since these methods are often limited, for instance to the linear regime or by the shape of the inhomogeneities that can be modelled, a different approach is necessary for the general nonlinear case and arbitrary shapes of the inhomogeneities. One possibility is given by computational homogenisation, which requires a nested solution scheme involving the computation of a boundary value problem (BVP) at the microscopic level, using a representative volume element (RVE) for every so-called material point.

Using the Finite Element Method to compute approximate solutions to the governing equations on both scales is often referred to as the $\text {FE}^2$-method and does not rely on macroscopic constitutive models, but on the solution of underlying BVPs and a consistent transition between the two scales [16]. This approach is usually accompanied by high computational costs, due to the repeated solution of numerous BVPs at the micro-scale in a possibly high dimensional space. One approach to lower the computational cost is given by methods that rely on the fast Fourier transformation (FFT) [28, 37]. These methods replace the assembly of the system tangent and the subsequent solution of the linear system on e.g. the micro-scale by an algorithm that utilizes FFT. This lowers the complexity of the solution process but does not necessarily reduce storage requirements [14].

Within this work, projection-based ROM is considered, which is characterised by taking advantage of the observation that the solutions of the aforementioned boundary value problems often lie in a lower dimensional subspace and different computational tasks in the offline and online stages. During the offline stage all necessary computations aiming to build a basis for the reduced-order model are carried out. This basis is used in the online stage to compute approximate solutions using a lower dimensional description. Several methods for model reduction exist, e.g. Proper Generalized Decomposition [8, 10], Reduced-Basis methods [3, 30, 31], or approaches using the Proper Orthogonal Decomposition (POD). The latter has been widely used, e.g. in homogenisation, fluid mechanics and many other fields [6, 17, 18, 23, 26, 41]. For a deeper discussion and a broader overview the reader is referred to [2, 33] and references therein.

In the context of computational homogenisation the Reduced Model Multiscale Method (R3M) has been presented in [41], applying POD-based model order reduction to the BVP on the micro-scale for the case of hyperelasticity, directly projecting the governing equations onto the truncated POD basis. Due to the missing approximation of the arising nonlinear terms the computational savings were limited. In general, the approach of solely applying e.g. Galerkin projection for nonlinear problems may even lead to higher computational cost compared to the Finite Element model.

A further contribution in the context of geometrically linear homogenisation is given by  [18], in which the stress field itself is approximated by a POD basis. Furthermore the authors showed in which circumstances an ill-posed system is obtained in the context of computational homogenisation and possibilities to avoid this problem. A similarly approach, i.e. not approximating the displacement field, is used in the nonuniform transformation field analysis (NTFA) [24], based on [11], and its extensions [14, 15, 25]. These methods apply a decomposition of the internal variables using reduced bases and derive suitable evolution equations for the reduced variables.

A popular approach in projection-based model reduction is the application of a Galerkin projection, as for e.g. the R3M, to solve the arising overdetermined system for the reduced coordinates. In the presence of nonlinearities these problems are often handled using an interpolation technique  [1, 7] or Gappy-POD reconstruction  [5, 13], both also often referred to as hyper-reduction techniques. Convergence difficulties have been reported for certain ROM configurations applying Galerkin projection coupled with hyper-reduction, e.g.  [5, 9, 22, 34]. In  [9] the condition number of the reduced system tangent is suspected to lead to said convergence problems. The authors therefore propose a gappy data reconstruction with Galerkin projection to improve the condition number. While improving the robustness the number of diverging ROMs has only been reduced. In [5] an alternative to the Galerkin projection is proposed, which is related to the Gauss-Newton algorithm (GNAT). This projection lowers the constraints of the arising system tangent matrix and renders a robust model reduction technique.

The aim of this contribution is to apply projection-based ROM techniques in the context of computational homogenisation of hyperelastic materials including hyper-reduction techniques, which requires a robust reduced model for the present multi-query context. The focus thereby lies on the problem on the micro-scale, i. e. the quantities computed on the micro-scale which would be used on the macro-scale in a fully coupled problem formulation. The main objective is to compare three different model reduction approaches in terms of accuracy, robustness and optimality.

The remainder of this contribution is organised as follows: Sect. 2 discusses the fundamentals of first-order computational homogenisation and the governing equations. Section 3 describes the ROM techniques used within this study, including considerations regarding optimality of different projection techniques. Section 4 presents numerical examples, followed by some concluding remarks in Sect. 5.

## Computational homogenisation in a nutshell

**Fig. 1:**
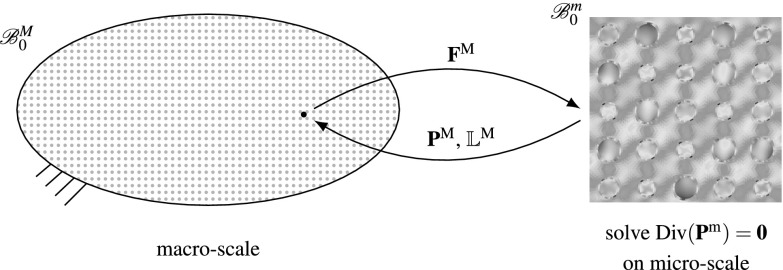
General concept of first-order computational homogenisation

In solid mechanics, phenomenological constitutive models are frequently used to describe the material response of a body under a given load. However, the response is mainly driven by the heterogeneous microstructure. One possible approach to account for the heterogeneous material would be to directly model the substructure, e. g. within a FE model, which usually leads to computationally expensive models. A different approach is given by computational homogenisation. In this context the material at the macroscopic level is modelled without a prescribed constitutive model. Instead, the constitutive response is computed for every so-called material point at the micro-scale, taking into account the heterogeneities through prescribed constitutive behaviour of the constituents. To distinguish between the two scales, the superscripts $(\bullet )^\mathbf {M}$ and $(\bullet )^\mathbf {m}$ are used to denote quantities on the macro- and micro-scale respectively. Figure 1 illustrates the general concept of this method.

On both scales the balance of linear momentum represents the equation of interest, which reads on the micro-scale

2.1 $$\begin{aligned} {\mathrm{Div}}\mathbf {P}^\mathbf {m}= {\mathbf {0}} \text { in } \mathscr {B}_0^m , \end{aligned}$$

where $\mathbf {P}^\mathbf {m}$ denotes the Piola stress on the micro-scale and $\mathscr {B}_0^m$ the domain of the RVE. The microscopic stresses are computed using a hyperelastic constitutive model, i. e.

2.2 $$\begin{aligned} \mathbf {P}^\mathbf {m}= \frac{\partial \varPsi ^{\mathbf {m}} (\mathbf {F^m})}{\partial \mathbf {F^m}}, \end{aligned}$$

with $\mathbf {F^m}$ representing the micro-scale deformation gradient and $\varPsi ^{\mathbf {m}}$ the strain energy density. The material response on the macro-scale relies on quantities computed on the micro-scale. In order to compute these quantities, certain requirements have to be satisfied.

### Hill-Mandel condition

A main ingredient of scale transition is the equality of the virtual work on the macro- and micro-scale, known as Hill-Mandel condition [19–21],

2.3 $$\begin{aligned} \frac{1}{\text { V}_0} \int \limits _{\mathscr {B}_0^m } \mathbf {P}^\mathbf {m}: \delta \mathbf {F^m}\text { dV}= \mathbf {P^M}: \delta \mathbf {F^M}, \end{aligned}$$

where $\text { V}_0$ denotes the volume of the RVE in the reference configuration. Assuming a linear ansatz for the deformation on the micro-scale,

2.4 $$\begin{aligned} \mathbf {x}^\mathbf {m}= \mathbf {F^M}\cdot \mathbf {X}^\mathbf {m}+ {\tilde{\mathbf {u}}}, \end{aligned}$$

with ${\tilde{\mathbf {u}}}$ denoting the fluctuation field and $\mathbf {X}^\mathbf {m}$, $\mathbf {x}^\mathbf {m}$ the coordinates in the reference and spatial configuration respectively, the deformation gradient is given by

2.5 $$\begin{aligned} \mathbf {F^m}= \nabla _X\mathbf {x}^\mathbf {m}= \mathbf {F^M}+ \nabla _X{\tilde{\mathbf {u}}}. \end{aligned}$$

Considering the variation of the deformation gradient

2.6 $$\begin{aligned} \delta \mathbf {F^m}= \delta \mathbf {F^M}+ \nabla _X\delta {\tilde{\mathbf {u}}}\end{aligned}$$

and inserting Eq. (2.6) into Eq. (2.3) leads to

2.7 $$\begin{aligned} 0 =&\frac{1}{\text { V}_0} \int \limits _{\mathscr {B}_0^m } \mathbf {P}^\mathbf {m}: \left[ \delta \mathbf {F^M}+ \nabla _X\delta {\tilde{\mathbf {u}}}\right] \text { dV}- \mathbf {P^M}: \delta \mathbf {F^M}\\ =&\left[ \frac{1}{\text { V}_0} \int \limits _{\mathscr {B}_0^m } \mathbf {P}^\mathbf {m}\text { dV}- \mathbf {P^M}\right] : \delta \mathbf {F^M}\nonumber \\&+ \left[ \frac{1}{\text { V}_0} \int \limits _{\mathscr {B}_0^m } \mathbf {P}^\mathbf {m}: \nabla _X\delta {\tilde{\mathbf {u}}}\text { dV}\right] \nonumber . \end{aligned}$$

It follows that the volume average of the microscopic Piola stress has to equal its macro-scale counterpart, i. e.

2.8 $$\begin{aligned} \frac{1}{\text { V}_0} \int \limits _{\mathscr {B}_0^m } \mathbf {P}^\mathbf {m}\text { dV}= \mathbf {P^M}, \end{aligned}$$

for the first term to vanish. Furthermore, the second term, containing the fluctuations, has to vanish in order to comply with the Hill-Mandel condition. Reformulating this term as a boundary integral renders admissible boundary conditions. Within this work the fluctuations are set to vanish on the Dirichlet boundary, which yields linear displacement boundary conditions:

2.9 $$\begin{aligned} \mathbf {u}^{\mathbf {m}} = \left[ \mathbf {F^M}- \mathbf {1} \right] \cdot \mathbf {X}^\mathbf {m}\text { on } \partial \mathscr {B}_0^m \end{aligned}$$

It should be noted that alternatively periodic boundary conditions could be applied.

### Computation of the tangent

To provide information about the behaviour at a material point due to an increased load, the macroscopic tangent modulus has to be computed. In the present work the approach in  [39] is used. Therein, a series of numerical test cases is performed in order to compute the fourth order Lagrangian elasticity tensor

2.10 $$\begin{aligned} \mathbb {L}^{\text {M}}= \frac{\partial \mathbf {P^M}}{\partial \mathbf {F^M}} . \end{aligned}$$

This leads, depending on the dimension d of the problem, to a total of d$^2$ linear perturbation test cases at the converged equilibrium state with the perturbations of the macroscopic deformation gradient

2.11 $$\begin{aligned} \delta _{iJ} \mathbf {F^M}= h\; \mathbf {e}_i \otimes \mathbf {E}_J \; \text { with } 0< h \ll 1 , \end{aligned}$$

with $\mathbf {e}_i$ and $\mathbf {E}_J$ denoting the basis vectors in the spatial and the reference configuration respectively. Hence, the computations

2.12 $$\begin{aligned} \left[ \mathbb {L}^{\text {M}}\right] _{kLiJ} = \frac{\left[ \delta _{iJ} \mathbf {P^M}\right] _{kL}}{h} \end{aligned}$$

are necessary to compute the constitutive constants.

### Weak form and spatial discretisation

Within the scope of this contribution, an approximate solution to the micro-scale problem is computed using the FEM, which requires the weak formulation

2.13 $$\begin{aligned}&\int \nolimits _{\mathscr {B}_0^m } \nabla _X \delta \mathbf {u^m}: \mathbf {P}^\mathbf {m}\;\text { dV}= 0 ,\;\;\; \forall \delta \mathbf {u^m}\\&\text {with } \mathbf {u}^{\mathbf {m}} = \left[ \mathbf {F^M}- \mathbf {1} \right] \cdot \mathbf {X}^\mathbf {m}\text { on } \partial {\mathscr {B}_0^m } .\nonumber \end{aligned}$$

The discretisation of the above equations is carried out using the Bubnov-Galerkin Finite Element Method in a standard manner, i. e.

2.14 $$\begin{aligned} \mathbf {u}^{\mathbf {m},\text {el}} = \sum \limits _{i=1}^{n_{en}} \text {N}_i \mathbf {u}_i , \;\; \delta \mathbf {u}^{\mathbf {m},\text {el}} = \sum \limits _{i=1}^{n_{en}} \text {N}_i \delta \mathbf {u}_i , \end{aligned}$$

where the displacement field and the test function are approximated as a sum of shape functions (Lagrange polynomials) and nodal values, with $n_{en}$ denoting the number of element nodes. Using the definitions given in Eq. (2.14), the discretisation of Eq. (2.13) takes the form

2.15 $$\begin{aligned}&\int \limits _{\mathscr {B}_0^m } \nabla _X\delta \mathbf {u^m}: \mathbf {P}^\mathbf {m}\; \text { dV}\approx \nonumber \\&\quad \overset{n_{el}}{\underset{e=1}{\mathscr {A}}}\int \limits _{{\mathscr {B}_0^m }^{,e}} \sum \limits _{i=1}^{n_{en}} \left[ \delta \mathbf {u^m}_i \otimes \nabla _X\text {N}_i \right] : \mathbf {P}^\mathbf {m}\; \text { dV}^e = 0 , \end{aligned}$$

with the operator $\overset{n_{el}}{\underset{e=1}{\mathscr {A}}}$ representing the assembly of the element contributions. Since Eq. (2.15) has to hold for arbitrary $\delta \mathbf {u^m}_i$, it follows that

2.16 $$\begin{aligned} \overset{n_{el}}{\underset{e=1}{\mathscr {A}}}\int \limits _{{\mathscr {B}_0^m }^{,e}} \sum \limits _{i=1}^{n_{en}} \nabla _X\text {N}_i \cdot \mathbf {P}^\mathbf {m}\; \text { dV}^e = \mathbf {0} , \end{aligned}$$

which may be written as a vector-valued equation

2.17 $$\begin{aligned} \mathbf {f}^{\mathbf {m}} (\mathbf {u}^{\mathbf {m}})= \mathbf {0} . \end{aligned}$$

Hence, $\mathbf {f}^{\mathbf {m}}$ represents a nonlinear function of the unknown nodal displacement values. In order to find an approximate solution an iterative Newton-Raphson scheme is used. This requires the linearisation of the nonlinear function

2.18 $$\begin{aligned} \varDelta \mathbf {f}^{\mathbf {m}} = \mathbf {K}^{\mathbf {m}} \cdot \varDelta \mathbf {u}^{\mathbf {m}} , \end{aligned}$$

which yields the tangent stiffness matrix

2.19 $$\begin{aligned} \mathbf {K}^{\mathbf {m}}_{IJ} = \overset{n_{el}}{\underset{e=1}{\mathscr {A}}}\left[ \int \nolimits _{{\mathscr {B}_0^m }^{,e}} \left[ \nabla _X\text {N}_i \cdot \frac{\partial \mathbf {P}^\mathbf {m}}{\partial \mathbf {F^m}} \cdot \nabla _X\text {N}_j \right] \text { dV}^e \right] . \end{aligned}$$

Starting at an initial guess of the solution $\mathbf {u}^{\mathbf {m},k}$, an iterative update of the displacement is computed via

2.20 $$\begin{aligned} \varDelta \mathbf {u}^{\mathbf {m},k} = - \left[ \mathbf {K}^{\mathbf {m},k}\right] ^{-1} \cdot {\mathbf {f}}^{\mathbf {m},k} , \end{aligned}$$

leading to

2.21 $$\begin{aligned} \mathbf {u}^{{\mathbf {m}},k+1} = \mathbf {u}^{{\mathbf {m}},k} + \varDelta \mathbf {u}^{\mathbf {m},k} . \end{aligned}$$

This iterative procedure is repeated until a prescribed convergence criterion is satisfied. Since the focus lies on the micro-scale problem, the superscripts $\left( \bullet \right) ^{\mathbf {m}}$ will be omitted in the following in order to improve readability and only be used if it is required in the context. Furthermore, Eq. (2.17) will be solved for the unknown fluctuation field ${\tilde{\mathbf {u}}}$ instead for the micro displacement field $\mathbf {u}$.

## Reduced-order modelling

As presented in the previous section, one possibility to compute approximate solutions to the microscopic problem defined by Eq. (2.1) involves the use of the Finite Element Method. Depending on the discretisation of the considered domain this may lead to large-scale systems. Especially in a multi-query context, as is the case in computational homogenisation, this leads to high computational costs. The solutions of such systems often lie in an affine subspace of lower dimension and therefore techniques to reduce the dimensionality of such problems are desired. Within the scope of this study a POD-based ROM approach is applied, including various hyper-reduction techniques.

### Proper Orthogonal Decomposition

Consider discrete values ${\mathbf {a}_{\left( \bullet \right) }}_j$, e.g. displacement fluctuation values of the BVP of the micro-scale problem, solved using the Finite Element Method. These so-called snapshots are arranged into a matrix $\mathbf {A}$

3.1 $$\begin{aligned} \mathbf {A}_{\left( {\tilde{\mathbf {u}}}\right) } = \left[ {\mathbf {a}_{\left( {\tilde{\mathbf {u}}}\right) }}_1,...,{\mathbf {a}_{\left( {\tilde{\mathbf {u}}}\right) }}_{n_s} \right] \in \mathbb {R}^{n \times n_s} , \end{aligned}$$

with rank $d \le \text {min}(n,n_s)$, *n* denoting the number of degrees of freedom of the FEM model and $n_s$ the number of snapshots. These solutions span a certain space denoted by $\mathscr {V}$. The snapshot POD [36] is then used to filter out the dominant characteristics, allowing the computation of an orthonormal basis, that best suites a rank $l \ll d$ approximation of the snapshots in a least-squares sense. This task can be formulated as a constraint optimization problem. It can be shown that the solution of this optimization problem is given by the first *l* left singular vectors $\mathbf {U}\left( :,1:l\right)$ of $\mathbf {A}_{\left( {\tilde{\mathbf {u}}}\right) } = \mathbf {U \cdot \varvec{\varSigma } \cdot \mathbf {V}^T}$ called the POD basis of rank *l* [2, 40]. The basis vectors optimally represent the snapshots in a least-squares sense for the given rank *l* approximation. For the choice of a suitable *l*, it is useful to consider a truncation criterion $\varepsilon$ in order to select the first *l* POD modes. For a system of rank *d* the criterion may be defined in terms of the singular values $\sigma _i$ as

3.2 $$\begin{aligned} \frac{\sum \limits _{i=1}^{l} \sigma _i^2}{\sum \limits _{i=1}^{d} \sigma _i^2} \ge 1 - \varepsilon , \end{aligned}$$

which gives information about the ability of the truncated basis to reproduce the snapshots. In the following, POD bases will be abbreviated by $\mathbf {U}^{\text {r}}_{\left( \bullet \right) }$, e.g. $\mathbf {U}^{\text {r}}_{\left( {\tilde{\mathbf {u}}}\right) } \in \mathbb {R}^{n \times l}$ for an *l*-dimensional POD basis of the displacement fluctuation field ${\tilde{\mathbf {u}}}$. In case of large snapshot sets, a nested POD as given in  [4, 32] may be used, which in essence partitions the snapshots into smaller sets, computes a lower rank approximation of each set and eventually computes a POD of the low rank approximations of the snapshot sets.

### Projection approaches

Introducing the dimensionality reduction for the primary unknown via the POD renders an overdetermined system of equations and therefore suitable projection techniques are required. The widely used Galerkin projection and an alternative Petrov-Galerkin projection are briefly reviewed in this section. For a more detailed discussion the reader is referred to [5, 35].

#### Galerkin projection

Employing the Galerkin projection the fluctuation field ${\tilde{\mathbf {u}}}$ and the test function are approximated using the POD basis vectors, i. e.

3.3 $$\begin{aligned} {\tilde{\mathbf {u}}}= \underset{n \times l}{\underbrace{\mathbf {U}^{\text {r}}_{\left( {\tilde{\mathbf {u}}}\right) }}} \cdot \underbrace{\hat{\mathbf {u}}}_{l \times 1} \; \text { and }\; \delta \mathbf {u} = \underset{n \times l}{\underbrace{\mathbf {U}^{\text {r}}_{\left( {\tilde{\mathbf {u}}}\right) }}} \cdot \underbrace{\delta \hat{\mathbf {u}}}_{l \times 1} , \end{aligned}$$

with the generalised coordinates $\hat{\mathbf {u}}$ for the reduced model. Inserting the definitions from Eq. (3.3) into Eq. (2.17) renders the reduced nonlinear term

3.4 $$\begin{aligned} \hat{\mathbf {f}}= \underbrace{{\mathbf {U}^{\text {r}}_{\left( {\tilde{\mathbf {u}}}\right) }}^{\mathbf {T}}}_{l \times n} \cdot \underbrace{\mathbf {f}\left( \mathbf {U}^{\text {r}}_{\left( {\tilde{\mathbf {u}}}\right) } \cdot \hat{\mathbf {u}}\right) }_{n \times 1} \end{aligned}$$

and the corresponding reduced tangent stiffness matrix

3.5 $$\begin{aligned} \hat{\mathbf {K}}= \underbrace{{\mathbf {U}^{\text {r}}_{\left( {\tilde{\mathbf {u}}}\right) }}^{\mathbf {T}}}_{l \times n} \cdot \underbrace{\mathbf {K}\left( \mathbf {U}^{\text {r}}_{\left( {\tilde{\mathbf {u}}}\right) } \cdot \hat{\mathbf {u}}\right) }_{n \times n} \cdot \underbrace{{\mathbf {U}^{\text {r}}_{\left( {\tilde{\mathbf {u}}}\right) }}}_{n \times l} . \end{aligned}$$

Recalling the dimension of the POD basis matrix $\mathbf {U}^{\text {r}}_{\left( {\tilde{\mathbf {u}}}\right) } \in \mathbb {R}^{n \times l}$, with *n* equal to the number of degrees of freedom and *l* the number of selected modes according to a chosen error criterion, the obvious benefit of this procedure is that now a system of equations for only *l* unknowns has to be solved. This decreases the computational cost especially for $l \ll n$. Using the iterative Newton-Raphson scheme

3.6 $$\begin{aligned} \varDelta \hat{\mathbf {u}}= - \left[ {\hat{\mathbf {K}}}^k\right] ^{-1} \cdot {\hat{\mathbf {f}}} \end{aligned}$$

leads to the updated approximate solution

3.7 $$\begin{aligned} \hat{\mathbf {u}}^{k+1} = \hat{\mathbf {u}}^k + \varDelta \hat{\mathbf {u}}. \end{aligned}$$

As shown in  [5, 35] this projection, which produces Eq. (3.6), is optimal in the sense that it minimizes the error between the solutions of the reduced and the full order model in the $\mathbf {K}$-norm:

3.8 $$\begin{aligned} \varDelta \hat{\mathbf {u}}= {\text {arg }\underset{ \mathbf {w} \in \mathbb {R}^{l}}{\text {min}}} \left\| \mathbf {U}^{\text {r}}_{\left( {\tilde{\mathbf {u}}}\right) } \cdot \mathbf {w} - \left[ - \mathbf {K}^{-1} \cdot \mathbf {f}\right] \right\| _{\mathbf {K}} \end{aligned}$$

Thereby the tangent stiffness matrix has to be symmetric positive definite. It will be shown in Sect. 3.3 that neither of the hyper-reduction techniques discussed in this work guarantees the symmetry of the unreduced tangent stiffness matrix given in Eq. (3.20). Hence, the Galerkin projection combined with the hyper-reduction approaches as discussed within the work is not optimal in the sense of Eq. (3.8).

#### Petrov-Galerkin projection

As shown in Eq. (3.4) and Eq. (3.5) the Galerkin projection multiplies ${\mathbf {U}^{\text {r}}_{\left( {\tilde{\mathbf {u}}}\right) }}^{\mathbf {T}}$ from the left. An alternative approach is given by selecting ${\left[ \mathbf {K}\cdot \mathbf {U}^{\text {r}}_{\left( {\tilde{\mathbf {u}}}\right) } \right] }^{\mathbf {T}}$ to be multiplied from the left, which results in

3.9 $$\begin{aligned} \hat{\mathbf {f}}= \underbrace{ {\mathbf {U}^{\text {r}}_{\left( {\tilde{\mathbf {u}}}\right) }}^{\mathbf {T}}{\mathbf {K}}^{\mathbf {T}}}_{l \times n} \cdot \underbrace{ \mathbf {f}}_{n \times 1} \end{aligned}$$

and

3.10 $$\begin{aligned} \hat{\mathbf {K}}= \underbrace{{\mathbf {U}^{\text {r}}_{\left( {\tilde{\mathbf {u}}}\right) }}^{\mathbf {T}}{\mathbf {K}}^{\mathbf {T}}}_{l \times n} \cdot \underbrace{\mathbf {K}\left( \mathbf {U}^{\text {r}}_{\left( {\tilde{\mathbf {u}}}\right) } \cdot \hat{\mathbf {u}}\right) }_{n \times n} \cdot \underbrace{{\mathbf {U}^{\text {r}}_{\left( {\tilde{\mathbf {u}}}\right) }}}_{n \times l} . \end{aligned}$$

This approach renders

3.11 $$\begin{aligned} \varDelta \hat{\mathbf {u}}= \text {arg } {\underset{\mathbf {w} \in \mathbb {R}^l}{\text {min}}} \left\| \mathbf {U}^{\text {r}}_{\left( {\tilde{\mathbf {u}}}\right) } \cdot \mathbf {w} - \left[ - \mathbf {K}^{-1} \cdot \mathbf {f}\right] \right\| _{\mathbf {K}^{\text {T}}\mathbf {K}} \end{aligned}$$

and corresponds to the least-square problem

3.12 $$\begin{aligned} \varDelta \hat{\mathbf {u}}= \text {arg } {\underset{\mathbf {w} \in \mathbb {R}^l}{\text {min}}} \left\| \mathbf {K}\cdot \mathbf {U}^{\text {r}}_{\left( {\tilde{\mathbf {u}}}\right) } \cdot \mathbf {w} + \mathbf {f}\right\| _{\varvec{2}}, \end{aligned}$$

requiring the tangent stiffness matrix solely to be regular [5, 35].

While reducing the number of unknowns, the nonlinear terms still have to be evaluated at the full scale and projected onto the subspace at every iteration step, which clearly limits the computational savings. Hence, further reduction techniques have to be applied in order to significantly reduce the computational cost.

### Hyper-reduction

As previously highlighted, the direct projection approach still depends on the full scale dimension *n*, due to the evaluation of the nonlinear terms. There exists a variety of approximation techniques for nonlinearities such as Empirical Interpolation Method (EIM) [1], its extension Discrete Empirical Interpolation Method (DEIM) [7] or the Gappy-POD  [5, 13], amongst others. It should be noted that, as shown in  [18], employing hyper-reduction may lead to ill-posed systems, since the internal force vector, which is approximated using hyper-reduction, is zero at a converged state. Within our studies the bases for the subsequent hyper-reduction techniques are computed using snapshots of the internal force vector during the iterative solution process (the vector is non-zero). Hence, using a non-truncated basis for the approximated nonlinearity, one obtains the same internal force vector as that of the full order model, which justifies this approach. Within the present study the DEIM and the Gappy-POD in combination with the discussed projection approaches are compared and will therefore be shortly discussed.

#### Discrete empirical interpolation method

In essence, this method approximates a nonlinear function as

3.13 $$\begin{aligned} \mathbf {f}\left( {\tilde{\mathbf {u}}}\left( \mu \right) \right) \approx \mathbf {U}^{\text {r}}_{\left( \mathbf {f}\right) } \cdot \mathbf {c}\left( {\tilde{\mathbf {u}}}\left( \mu \right) \right) , \end{aligned}$$

where the parameter $\mu$ is introduced to denote the dependence of the fluctuation field on the macroscopic deformation gradient $\mathbf {F^M}$, used to compute the macroscopic displacement field. The parameter stems from a suitable parameter space $\mu \in \mathscr {D}\subset \mathbb {R}^d$, e. g. $\mu = \left[ \text {F}^{\text {M}}_{11} , \text {F}^{\text {M}}_{12}, \text {F}^{\text {M}}_{21}, \text {F}^{\text {M}}_{22} \right] \in \mathscr {D}\subset \mathbb {R}^4$, for the two dimensional case. The direct projection approach requires the collection of snapshots ${\mathbf {a}_{\left( {\tilde{\mathbf {u}}}\right) }}_i$ of the Finite Element approximated fluctuation field arranged into $\mathbf {A}_{\left( {\tilde{\mathbf {u}}}\right) }$ in order to compute the POD basis. Using DEIM, snapshots of the corresponding nonlinear function ${\mathbf {a}_{\left( \mathbf {f}\right) }}_i$, see Eq. (2.17), are collected during the iterative solution procedure in the offline phase and assembled into $\mathbf {A}_{\left( \mathbf {f}\right) }$,

3.14 $$\begin{aligned} \mathbf {A}_{\left( \mathbf {f}\right) } = \left[ {\mathbf {a}_{\left( \mathbf {f}\right) }}_1,...,{\mathbf {a}_{\left( \mathbf {f}\right) }}_{n_s} \right] , \end{aligned}$$

where $n_s$ equals the number of considered snapshots. Performing the POD of $\mathbf {A}_{\left( \mathbf {f}\right) }$ renders the matrix $\mathbf {U}^{\text {r}}_{\left( \mathbf {f}\right) } \in \mathbb {R}^{n \times k}$, representing a *k*-dimensional orthonormal basis, i.e. *k* modes are considered, for the space spanned by the snapshots of the nonlinear term. The coefficients of $\mathbf {c}$ in Eq. (3.13) are computed using *k* rows of $\mathbf {f}\left( {\tilde{\mathbf {u}}}\left( \mu \right) \right)$

3.15 $$\begin{aligned} \mathscr {P}^{\mathbf {T}}\cdot \mathbf {f}= \left[ \mathscr {P}^{\mathbf {T}}\cdot \mathbf {U}^{\text {r}}_{\left( \mathbf {f}\right) } \right] \cdot \mathbf {c}\end{aligned}$$

where $\mathscr {P}$ denotes an extraction operator. This may be considered as a matrix composed of *k* vectors

3.16 $$\begin{aligned} \mathscr {P}= \left[ \mathbf {i}_{{\rho }_1}, ... , \mathbf {i}_{{\rho }_k} \right] \in \mathbb {R}^{n \times k} , \end{aligned}$$

where $\mathbf {i}_{{\rho }_i} = \left[ 0,...,0,1,0,...,0 \right] ^{\mathbf {T}}$ denotes a vector in which the position of the only nonzero entry corresponds to the index $\rho _i$ [7]. Since the matrix $\left[ \mathscr {P}^{\mathbf {T}}\cdot \mathbf {U}^{\text {r}}_{\left( \mathbf {f}\right) } \right]$ is always regular [7] the coefficients of $\mathbf {c}$ can be uniquely determined. This leads together with Eq. (3.15) to the DEIM approximation

3.17 $$\begin{aligned} \mathbf {f}\; \approx \; \mathbf {U}^{\text {r}}_{\left( \mathbf {f}\right) } \cdot \mathbf {c}\; = \; \underbrace{\mathbf {U}^{\text {r}}_{\left( \mathbf {f}\right) } \cdot \left[ \mathscr {P}^{\mathbf {T}}\cdot \mathbf {U}^{\text {r}}_{\left( \mathbf {f}\right) } \right] ^{-1}}_{n \times k} \cdot \underbrace{\mathscr {P}^{\mathbf {T}}\cdot \mathbf {f}}_{k \times 1} . \end{aligned}$$

The nonlinear term $\mathbf {f}$ now only needs to be evaluated at *k* entries specified by $\mathscr {P}$. The corresponding DEIM indices $\rho$ are determined using algorithm 1, proposed in [7], which computes the indices $\rho$ based on the basis $\mathbf {U}^{\text {r}}_{\left( \mathbf {f}\right) }$. The reduced nonlinear term reads after Galerkin projection

3.18 $$\begin{aligned} \hat{\mathbf {f}}\; = \; \underbrace{\left. \mathbf {U}^{\text {r}}_{\left( {\tilde{\mathbf {u}}}\right) }\right. ^{\mathbf {T}}\cdot \mathbf {U}^{\text {r}}_{\left( \mathbf {f}\right) } \cdot \left[ \mathscr {P}^{\mathbf {T}}\cdot \mathbf {U}^{\text {r}}_{\left( \mathbf {f}\right) } \right] ^{-1}}_{l \times k} \cdot \underbrace{\mathscr {P}^{\mathbf {T}}\cdot \mathbf {f}}_{k \times 1} , \end{aligned}$$

where the first term, of dimension $l \times k$, represents a constant quantity and is thus computed during the offline phase. Online only *k* components, corresponding to the *k* DEIM indices, need to be computed. The tangent is obtained as the derivative of Eq. (3.18),

3.19 $$\begin{aligned} \hat{\mathbf {K}}\; = \; \underbrace{\left. \mathbf {U}^{\text {r}}_{\left( {\tilde{\mathbf {u}}}\right) }\right. ^{\mathbf {T}}\cdot \mathbf {U}^{\text {r}}_{\left( \mathbf {f}\right) } \cdot \left[ \mathscr {P}^{\mathbf {T}}\cdot \mathbf {U}^{\text {r}}_{\left( \mathbf {f}\right) } \right] ^{-1}}_{l \times k} \cdot \underbrace{\mathscr {P}^{\mathbf {T}}\cdot \mathbf {K}\cdot \mathbf {U}^{\text {r}}_{\left( {\tilde{\mathbf {u}}}\right) }}_{k \times l}.\nonumber \\ \end{aligned}$$

The part of (3.19) which represents the tangent approximation,

3.20 $$\begin{aligned} \tilde{\mathbf {K}} \; = \; \underbrace{\mathbf {U}^{\text {r}}_{\left( \mathbf {f}\right) } \cdot \left[ \mathscr {P}^{\mathbf {T}}\cdot \mathbf {U}^{\text {r}}_{\left( \mathbf {f}\right) } \right] ^{-1}}_{n \times k} \cdot \underbrace{\mathscr {P}^{\mathbf {T}}\cdot \mathbf {K}}_{k \times n} , \end{aligned}$$

may not be symmetric as pointed out by  [5, 34]. Hence, applying a Galerkin projection is not optimal in the sense of Eq. (3.8). The same holds for the Gappy-POD in combination with a Galerkin projection. Consider therefore the following short example with the quantities

$$\begin{aligned} \mathbf {K}= \begin{bmatrix} 1&\quad 0&\quad 0&\quad 0 \\ 0&\quad 2&\quad -1&\quad 0 \\ 0&\quad -1&\quad 2&\quad 0 \\ 0&\quad 0&\quad 0&\quad 1 \end{bmatrix} ,\; \mathbf {U}^{\text {r}}_{\left( \mathbf {f}\right) } = \left[ \begin{array}{lll} 0 &{}\quad 0 &{}\quad \\ -0.7071 &{}\quad 0 \\ 0.7071 &{}\quad 0\\ 0 &{}\quad 1 \end{array}\right] \end{aligned}$$

and the sampling matrix

$$\begin{aligned} \mathscr {P}= \begin{bmatrix} 0&\quad 0&\quad \\ 1&\quad 0 \\ 0&\quad 0\\ 0&\quad 1 \end{bmatrix} . \end{aligned}$$

Using these matrices to evaluate Eq. (3.20) produces

3.21 $$\begin{aligned} \tilde{\mathbf {K}} = \begin{bmatrix} 0&\quad 0&\quad 0&\quad 0 \\ 0&\quad 2&\quad -1&\quad 0\\ 0&\quad -2&\quad 1&\quad 0 \\ 0&\quad 0&\quad 0&\quad 1 \end{bmatrix} , \end{aligned}$$

which is not symmetric. This small example shows that it can not be guaranteed that the tangent approximation in Eq. (3.20) preserves symmetry.
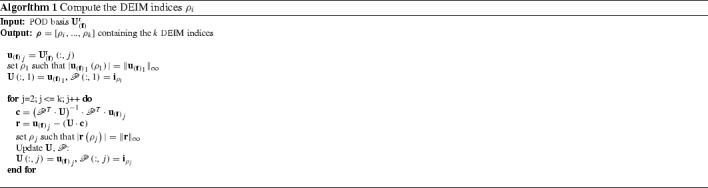


#### Gappy-POD

Contrary to the interpolation in Eq. (3.17) the Gappy-POD  [5, 9, 13] uses regression to approximate the nonlinear function. The approximation of the nonlinear term results in

3.22 $$\begin{aligned} \mathbf {f}&\approx \; \mathbf {U}^{\text {r}}_{\left( \mathbf {f}\right) } \cdot \mathbf {c}\; = \; \underbrace{\mathbf {U}^{\text {r}}_{\left( \mathbf {f}\right) } \cdot \left[ \mathscr {P}^{\mathbf {T}}\cdot \mathbf {U}^{\text {r}}_{\left( \mathbf {f}\right) } \right] ^{\dagger } }_{n \times k_s} \cdot \underbrace{\mathscr {P}^{\mathbf {T}}\cdot \mathbf {f}}_{k_s \times 1} . \end{aligned}$$

Here, $k_s$ indicates the number of sampling points with $k_s \ge k$, i.e. more sampling points than modes (keeping in mind that $\mathbf {U}^{\text {r}}_{\left( \mathbf {f}\right) } \in \mathbb {R}^{n \times k}$) and $\dagger$ denotes the pseudo-inverse. The tangent is computed analogously to Eq. (3.19). Similar to [5, 9], algorithm 2 represents the point selection algorithm used in this work.
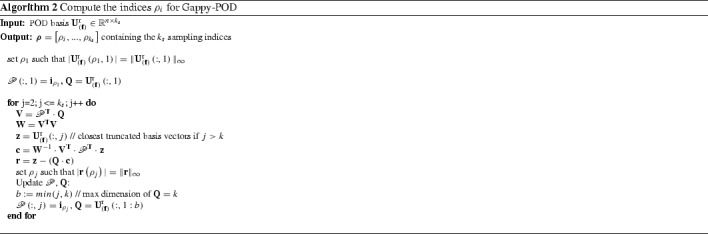


#### Gauss-Newton with approximated tensors (GNAT)

Instead of combining Galerkin projection and Gappy-POD, the GNAT solves the least-square problem in Eq. (3.12) using a Gappy-POD approximation of the nonlinear terms, which reads

3.23 $$\begin{aligned} \varDelta \hat{\mathbf {u}}= \text {arg } {\underset{\mathbf {w} \in \mathbb {R}^l}{\text {min}}} \left\| \mathbf {Y}\cdot \mathscr {P}^{\mathbf {T}}\cdot \mathbf {K}\cdot \mathbf {U}^{\text {r}}_{\left( {\tilde{\mathbf {u}}}\right) } \cdot \mathbf {w} + \mathbf {X}\cdot \mathscr {P}^{\mathbf {T}}\cdot \mathbf {f}\right\| _{2}\nonumber \\ \end{aligned}$$

with the matrices

$$\begin{aligned} \mathbf {X}&= {\mathbf {U}^{\text {r}}_{\left( \mathbf {K}\right) }}^{\mathbf {T}}\cdot \mathbf {U}^{\text {r}}_{\left( \mathbf {f}\right) } \cdot \left[ \mathscr {P}^{\mathbf {T}}\cdot \mathbf {U}^{\text {r}}_{\left( \mathbf {f}\right) } \right] ^{\dagger } \in \mathbb {R}^{k \times k_s} \\ \mathbf {Y}&= \left[ \mathscr {P}^{\mathbf {T}}\cdot \mathbf {U}^{\text {r}}_{\left( \mathbf {K}\right) } \right] ^{\dagger } \in \mathbb {R}^{k \times k_s} , \end{aligned}$$

using ${\mathbf {U}^{\text {r}}_{\left( \mathbf {K}\right) }}^{\mathbf {T}}\cdot {\mathbf {U}^{\text {r}}_{\left( \mathbf {K}\right) }} = \mathbf {I} \in \mathbb {R}^{k \times k}$, while being independent of the dimension of the FEM model *n*. Here, the quantity $\mathbf {U}^{\text {r}}_{\left( \mathbf {K}\right) }$ is introduced to account for the possibility of different snapshot selection strategies for the gappy approximation of the residual and the system tangent as presented in [5, 6]. Within the scope of the present work snapshots of the residual obtained from the Finite Element model (including the iterative states during the Newton-Raphson solution procedure) are used. These serve as the input to build the reduced basis for both the residual and the tangent, i.e. $\mathbf {U}^{\text {r}}_{\left( \mathbf {K}\right) } = \mathbf {U}^{\text {r}}_{\left( \mathbf {f}\right) }$.

## Numerical examples

**Fig. 2:**
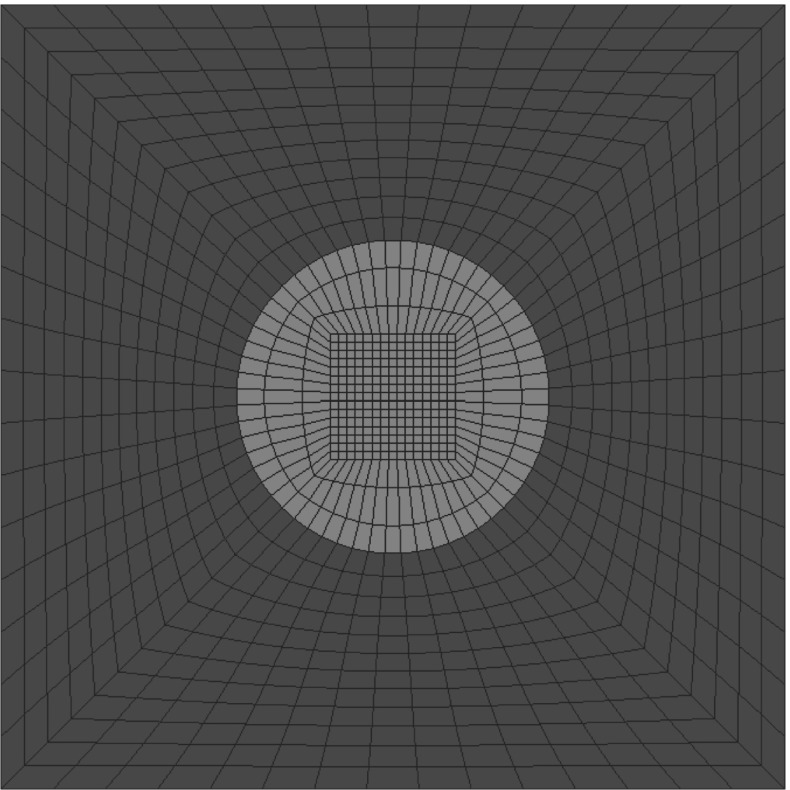
Finite Element model of fiber reinforced material

For the subsequent numerical examples a representative volume element of a Neo-Hookean hyperelastic material is used as depicted in Fig. 2. The matrix material and the inclusions differ in terms of the shear modulus , i.e. $\mu _{m}=3.4 \times 10^7$ and $\mu _{i}=2.0 \times 10^8$, while the Poisson’s ratio is set to be $\nu = 0.23$. The subscripts $\lbrace \bullet \rbrace _m$ and $\lbrace \bullet \rbrace _i$ denote the matrix and the inclusion respectively. The Finite Element discretisation renders of a total of 2.312 unknowns and the computations are carried out using a plane strain configuration.

As mentioned in Sect. 3 a snapshot POD is used to construct a reduced basis for the unknown fluctuation field. Hence, a training set $\mathscr {D}_{\text {train}}$ is necessary for which the full order model has to be computed. For the subsequent examples the training set was specified to be

4.1 $$\begin{aligned}&\mathscr {D}_{\text {train}}= \mathbf {1} + \varDelta \mathbf {F}^{\text {M}} \nonumber \\&\text {with } \left[ \varDelta \mathbf {F}^{\text {M}}\right] _{ij} \in \lbrace -0.2, -0.12, -0.04,\nonumber \\&\quad 0.04, 0.12, 0.2 \rbrace \end{aligned}$$

**Fig. 3:**
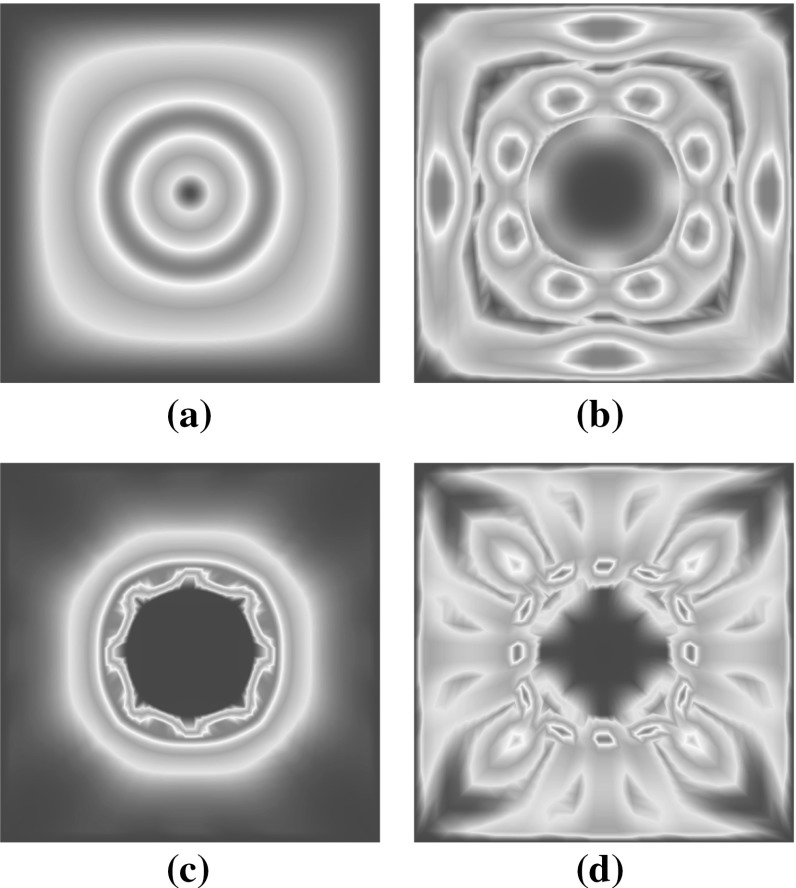
Examples for POD modes: **a** and **b** show the 1st and 19th mode of $\mathbf {U}^{\text {r}}_{\left( {\tilde{\mathbf {u}}}\right) }$ respectively; **c** and **d** show the 5th and $22{\text {th}}$ mode of $\mathbf {U}^{\text {r}}_{\left( \mathbf {f}\right) }$ respectively

Based on this training set a few POD basis modes for the fluctuation field and the nonlinear function are depicted in Fig. 3. One may observe the influence of taking snapshots of $\mathbf {f}$ during the iterative Newton-Raphson procedure where the internal force vector is non-zero.

### Robustness considerations

In this section the aforementioned different model reduction approaches, i.e. DEIM, Gappy-POD and GNAT are tested for various dimensionalities of $\mathbf {U}^{\text {r}}_{\left( {\tilde{\mathbf {u}}}\right) }$ and $\mathbf {U}^{\text {r}}_{\left( \mathbf {f}\right) }$ using the test set

4.2 $$\begin{aligned}&\mathscr {D}_{\text {test}}= \mathbf {1} + \varDelta \mathbf {F}^{\text {M}} \nonumber \\&\text {with } \left[ \varDelta \mathbf {F}^{\text {M}}\right] _{ij} \in \lbrace -0.1986, 0.1886, -0.1321,\nonumber \\&\quad 0.1461, -0.0521, 0.0921 \rbrace , \end{aligned}$$

which is different to the set $\mathscr {D}_{\text {train}}$. Therefore each configuration is tested against 1296 testcases. In case that any of said test cases does not converge using the reduced model the configuration is highlighted with an “x” in the subsequent result plots. Otherwise the color indicates the relative error of the fluctuation field in the $L_2$ norm

$$\begin{aligned} \epsilon ^{\text {rel}}_{{\tilde{\mathbf {u}}}} = \frac{ \Vert {\tilde{\mathbf {u}}}_{\text {FEM}} - {\tilde{\mathbf {u}}}_{\text {ROM}} \Vert _2 }{ \Vert {\tilde{\mathbf {u}}}_{\text {FEM}} \Vert _2}, \end{aligned}$$

averaged over the 1296 testcases. Furthermore the following measures are introduced:

$$\begin{aligned} \epsilon ^{\text {rel}}_{\mathbf {P^M}} = \frac{\left\| \mathbf {P^M}_{\text {FEM}} - \mathbf {P^M}_{\text {ROM}}\right\| _{\text {F}}}{\left\| \mathbf {P^M}_{\text {FEM}} \right\| _{\text {F}}} \;\;,\; \epsilon ^{\text {rel}}_{ \mathbb {L}^{\text {M}}} = \frac{\left\| \mathbb {L}^{\text {M}}_{\text {FEM}} - \mathbb {L}^{\text {M}}_{\text {ROM}}\right\| _{\text {F}}}{\left\| \mathbb {L}^{\text {M}}_{\text {FEM}} \right\| _{\text {F}}} \end{aligned}$$

**Fig. 4:**
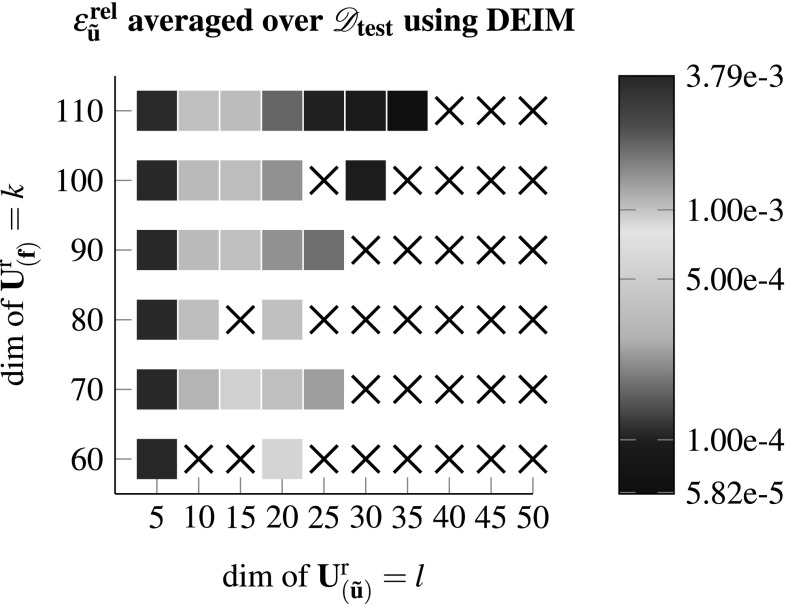
Using DEIM to compute $\mathscr {D}_{\text {test}}$ for various dimensions of the reduced bases. A “x” denotes the case were at least one test case within $\mathscr {D}_{\text {test}}$ did not converge

Figure 4 shows the results for the DEIM approach as described in Sect. 3.

One may observe that for various configurations the method rendered scenarios where at least one test case did not converge. In the multi-query context this clearly is an undesirable result as the model should return the quantities of interest for arbitrary input parameters. Considering the errors, they behave as expected and decrease for an increasing number of basis vector $\mathbf {U}^{\text {r}}_{\left( {\tilde{\mathbf {u}}}\right) }$. The influence of the dimension of $\mathbf {U}^{\text {r}}_{\left( \mathbf {f}\right) }$ appears to be rather small after a certain threshold, see also Figs. 8 and 9.

**Fig. 5:**
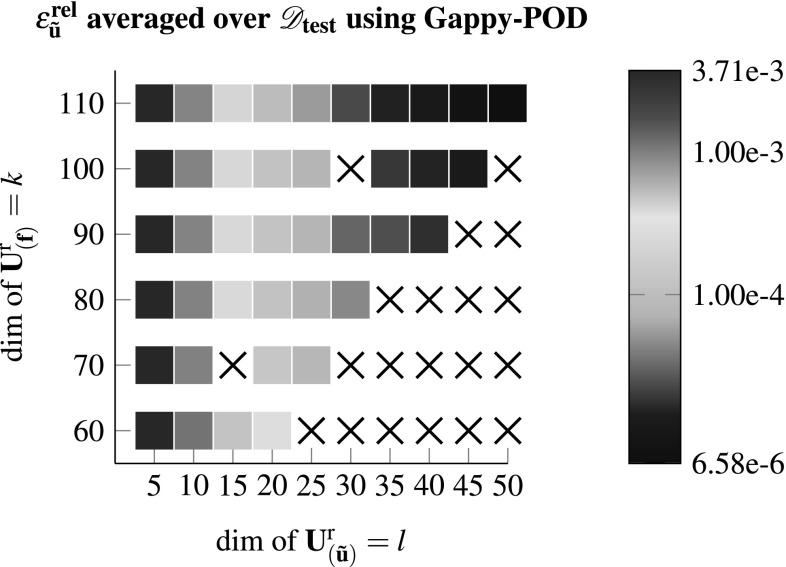
Using Gappy-POD with Galerkin projection to compute $\mathscr {D}_{\text {test}}$ for various dimensions of the reduced bases with ${k_s}/{k} = 2$. A “x” denotes the case were at least one test case within $\mathscr {D}_{\text {test}}$ did not converge

In  [9] it has been shown that using a Gappy-POD instead of pure interpolation benefits the condition number of the reduced tangent and should lead to a more robust model. This has also been observed within this work as shown in Fig. 5. While rendering a more robust model with respect to changes of the input parameters, as well as more accurate results, there were still configurations that lead to diverging test cases.

**Fig. 6:**
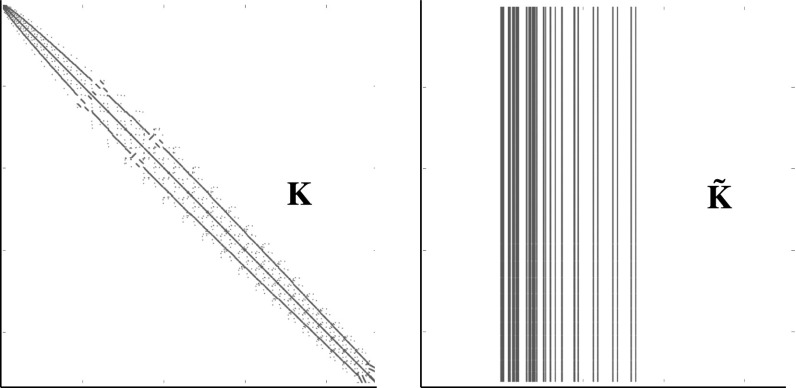
Sparsity Pattern of $\mathbf {K}$ and ${\tilde{\mathbf {K}}}$ (with $k=10$ and rank $\left( {\tilde{\mathbf {K}}}\right) = 10$)

**Fig. 7:**
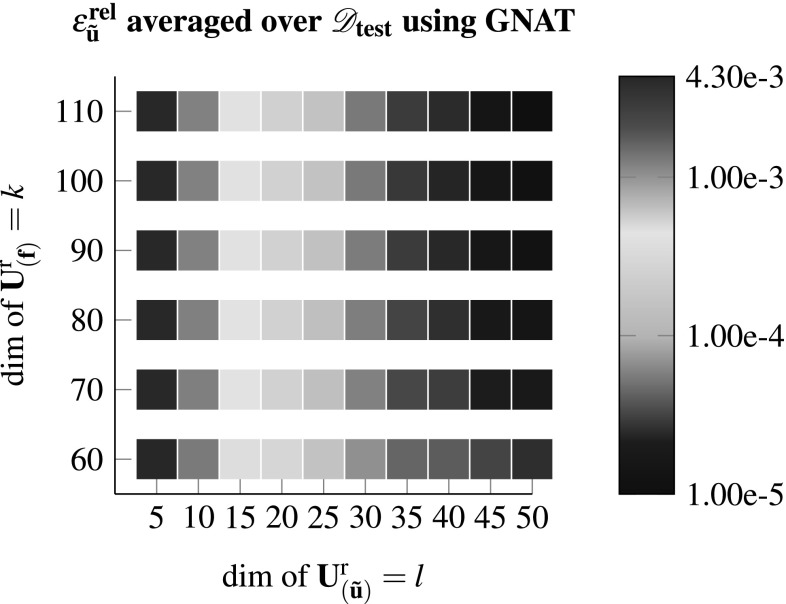
Using GNAT to compute $\mathscr {D}_{\text {test}}$ for various dimensions of the reduced bases with ${k_s}/{k} = 2$. A “x” denotes the case were at least one test case within $\mathscr {D}_{\text {test}}$ did not converge

This might be due to a possible loss of symmetry through the application of a hyper-reduction method and the subsequent non-optimal Galerkin projection. To illustrate the asymmetry of ${\tilde{\mathbf {K}}}$ a comparison of the sparsity patterns for the given example from Fig. 2 is shown in Fig. 6.

Employing therefore the GNAT, suited for non-symmetric tangent matrices, lead to the most robust model within the scope of this study as shown in Fig. 7.

**Fig. 8:**
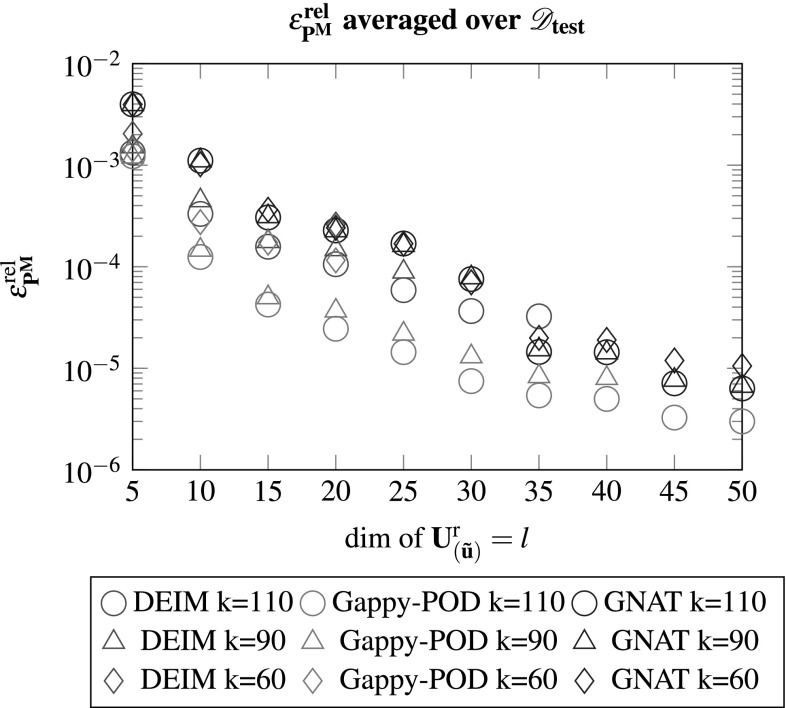
The relative error of the averaged Piola stress tensor computed by the FEM and the reduced model for an increasing number of *l* modes of $\mathbf {U}^{\text {r}}_{\left( {\tilde{\mathbf {u}}}\right) }$. A missing marker denotes a ROM with at least one diverged test case of $\mathscr {D}_{\text {test}}$ (see Figs. 4–7)

**Fig. 9:**
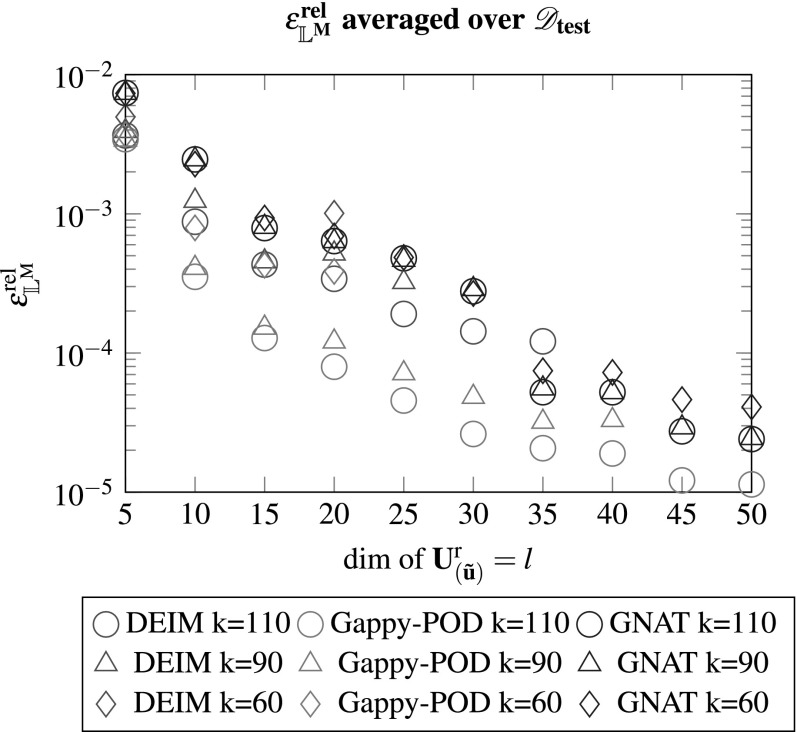
The relative error of the macroscopic tangent computed by the FEM and the reduced model for an increasing number of *l* modes of $\mathbf {U}^{\text {r}}_{\left( {\tilde{\mathbf {u}}}\right) }$. A missing marker denotes a ROM with at least one diverged test case of $\mathscr {D}_{\text {test}}$ (see Figs. 4–7)

One can observe that no configuration lead to a diverging test case and the method appears to be very robust. Furthermore, equivalently for DEIM and Gappy-POD, increasing the dimension of $\mathbf {U}^{\text {r}}_{\left( {\tilde{\mathbf {u}}}\right) }$ decreases the error, while a change of the dimensionality of $\mathbf {U}^{\text {r}}_{\left( \mathbf {f}\right) }$ has only minor effect on the accuracy above a certain threshold. This gets confirmed considering Figs. 8 and 9 which depict the errors for the averaged stresses and the macroscopic tangent.

Employing Gappy-POD with Galerkin projection rendered the most accurate results while the GNAT rendered the most robust model and showed no convergence difficulties. Though the Gappy-POD is more robust compared to the DEIM, there is no guarantee that a different $\mathscr {D}_{\text {test}}$ would yield the same behaviour. It should be noted that the reduced model has been used for the perturbation procedure from Sect. 2.2 to obtain the macroscopic tangent components while the stresses were averaged over the full domain using the solution of the reduced model $\mathbf {U}^{\text {r}}_{\left( {\tilde{\mathbf {u}}}\right) } \cdot \hat{\mathbf {u}}$.

**Fig. 10:**
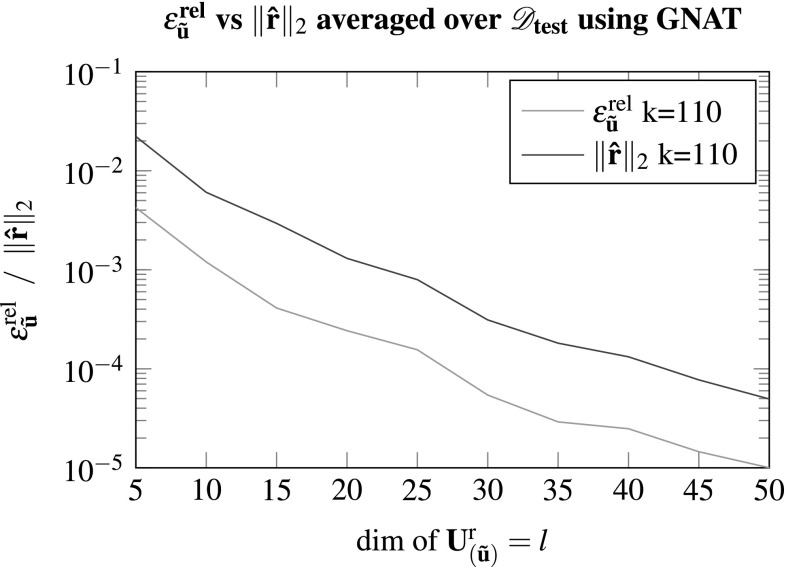
The reduced residual (${\hat{\mathbf {r}}} = - \mathbf {X}\cdot \mathscr {P}^{\mathbf {T}}\cdot \mathbf {f}$) from GNAT versus the relative error of the fluctuation field $\epsilon ^{\text {rel}}_{{\tilde{\mathbf {u}}}}$ with ${k_s}/{k} = 2$; for every *l* the error is averaged over all test cases within $\mathscr {D}_{\text {test}}$

A further benefit of GNAT is that it minimizes the global full order residual. Therefore using the reduced residual ${\hat{\mathbf {r}}} = - \mathbf {X}\cdot \mathscr {P}^{\mathbf {T}}\cdot \mathbf {f}$ from Eq. (3.23) may serve as an error indicator, considering the relation to the relative error as depicted in Fig. 10. The reduced residual as well as the relative error decrease with an increasing size of the reduced basis and match quite well up to a certain offset. This may be beneficial considering greedy selection algorithms to build the ROM, which rely on the ability to estimate or at least indicate the error of the reduced model approximation.

**Fig. 11:**
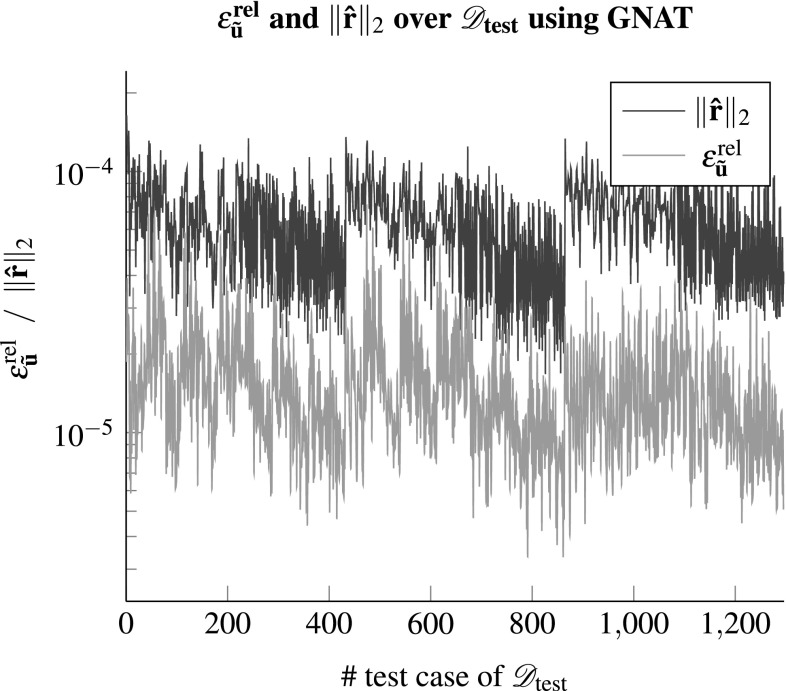
The norm of the reduced residual compared to the relative error of the fluctuation field for all test cases within $\mathscr {D}_{\text {test}}$ using GNAT with $l=45, k=90$ and ${k_s}/{k}=2$

The relation between the errors $\epsilon ^{\text {rel}}_{{\tilde{\mathbf {u}}}}$ and the residual is further investigated for a given ROM and all test cases of the parameter set $\mathscr {D}_{\text {test}}$ in Fig. 11. Based on the previous results the ROM dimensions are set to $l=45, k=90$ and ${k_s}/{k}=2$ using GNAT. It can be seen that the relative error has minor fluctuations but does not show considerable deviations from its mean. The same holds for the norm of the reduced residual from the GNAT model. Hence, the norm of the reduced residual appears to be a computational cheap and reliable error indicator which could be used in future studies in conjunction with an adaptive sampling algorithm, e.g. [32], instead of using $\mathscr {D}_{\text {train}}$ to build the reduced model.

**Fig. 12:**
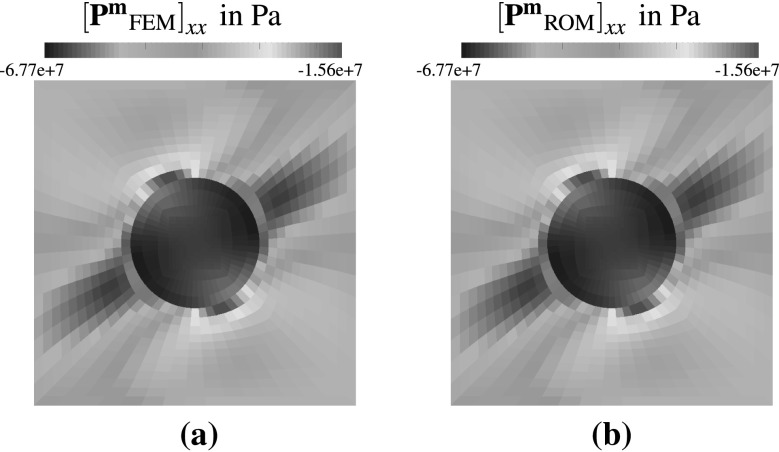
In **a** and **b** the xx-components of the Piola stress tensor (computed using the full order model and the ROM) are shown

### Local fields

Besides acting as a simple input-output model it is possible in computational homogenisation to investigate local fields. Considering a test case with

**Fig. 13:**
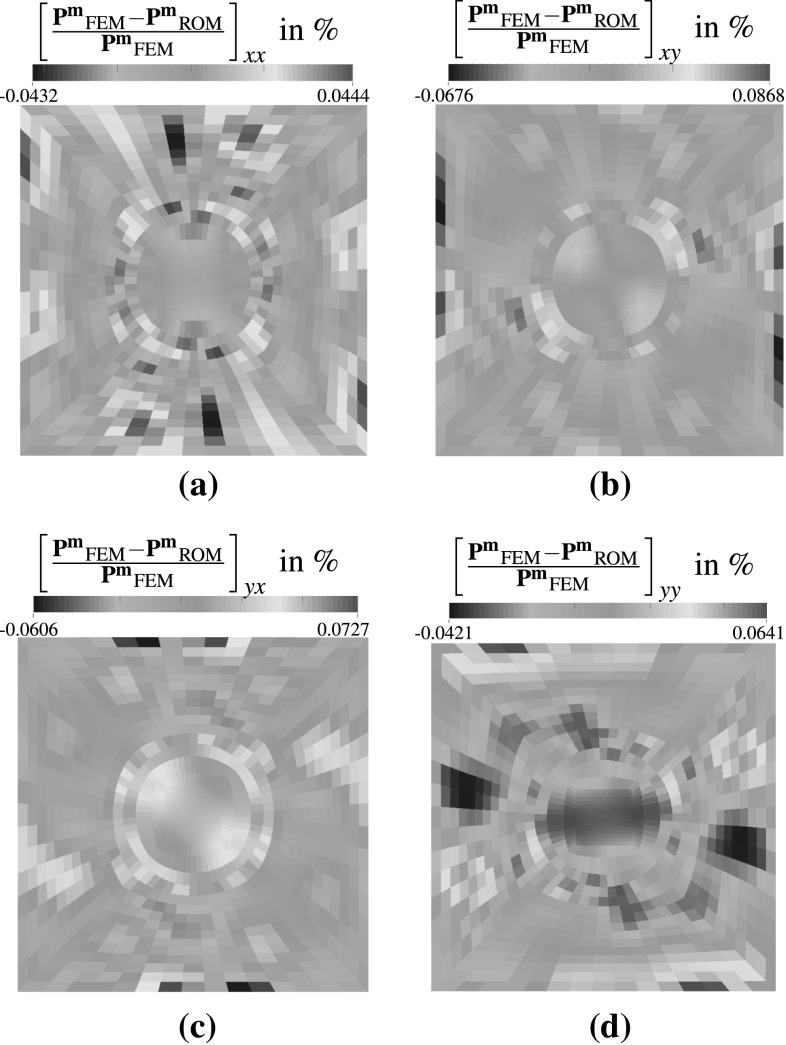
Figures **a**–**d** show the relative error of the individual stress components in percent

4.3 $$\begin{aligned} \mathbf {F}^{\text {M}} = \left( \begin{matrix} -1.1986 &{} -0.1986 \\ -0.1986 &{} -1.1986 \end{matrix} \right) \end{aligned}$$

the Piola stress components computed by the FEM and the reduced model are compared in Fig. 12 for the GNAT with $l=45, k=90$ and ${k_s}/{k}=2$. Hence, instead of solving for 2.312 unknowns only 45 unknowns have to be determined reducing the computational cost considerably. It can be seen that the xx-component of $\mathbf {P}^\mathbf {m}$ computed by the FEM model in Fig. 12a and the GNAT in Fig. 12b match quite well which is supported considering the relative errors in percent of the individual components of the stress tensor given in Fig. 13a–d. For the current ROM configuration the errors appear to be acceptable small and correlate with the observations in all the error plots throughout this section.

## Conclusion

Within the scope of the present work reduced-order modelling techniques based on the Proper Orthogonal Decomposition and so-called hyper-reduction techniques have been applied in the context of computational homogenisation of hyperelastic materials. The focus has thereby been the accuracy and robustness of the reduced model, comparing different hyper-reduction and projection approaches. It has been shown that introducing an additional approximation of the nonlinear terms via an empirical interpolation or Gappy-POD may not preserve the symmetry of the system tangent. This leads to a non-optimal Galerkin projection. As shown in the numerical examples this can cause convergence problems. This is clearly an undesirable property in the multi-query context as given in computational homogenisation. The Gauss-Newton like approach (GNAT), relying on a Petrov-Galerkin projection suited for non-symmetric tangent matrices, rendered the most robust model and showed no convergence problems while minimizing the global residual.

Future studies should aim towards more effective methods to obtain error estimates for the outputs of interest and averaging procedures. Furthermore, alternative approximations of the system tangent, e.g. [5, 29, 38], could be investigated. Yet some of these methods require additional high dimensional snapshots of the sparse system tangent which becomes computational infeasible rapidly. For instance considering only the non-zero elements of the tangent from the presented examples in Sect. 4 renders an additional snapshot of the size 40.836 for the 2.312 unknowns in every iteration step.

Regarding the snapshot selection adaptive strategies, e.g. [32], could be employed to adaptively select the positions in parameter space for which the full model should be evaluated in order to build the ROM.
